# The association between frailty and severe disease among COVID-19 patients aged over 60 years in China: a prospective cohort study

**DOI:** 10.1186/s12916-020-01761-0

**Published:** 2020-09-07

**Authors:** Yao Ma, Lisha Hou, Xiufang Yang, Zhixin Huang, Xue Yang, Na Zhao, Min He, Yixin Shi, Yan Kang, Jirong Yue, Chenkai Wu

**Affiliations:** 1 Department of Geriatrics and National Clinical Research Center for Geriatrics, West China Hospital, Sichuan University, Chengdu, Sichuan Province China; 2 COVID-19 Medical Assistance Teams (Hubei) of West China Hospital, Sichuan University, Chengdu, Sichuan Province China; 3 Mental Health Centre, West China Hospital, Sichuan University, Chengdu, Sichuan Province China; 4 Department of Obstetrics and Gynecology, Renmin Hospital of Wuhan University, Wuhan, Hubei Province China; 5 Department of Otolaryngology-Head and Neck Surgery, Renmin Hospital of Wuhan University, Wuhan, Hubei Province China; 6 Department of Critical Care Medicine, West China Hospital, Sichuan University, Chengdu, Sichuan Province China; 7 State Key Laboratory of Oral Diseases & National Clinical Research Center for Oral Diseases & West China School of Stomatology, Sichuan University, Chengdu, Sichuan Province China; 8 Global Health Research Center Duke Kunshan University, Suzhou, Jiangsu Province China

**Keywords:** Frailty, COVID-19, Severe disease, Prospective study, Older

## Abstract

**Background:**

The coronavirus disease 2019 (COVID-19) has been a pandemic worldwide. Old age and underlying illnesses are associated with poor prognosis among COVID-19 patients. However, whether frailty, a common geriatric syndrome of reduced reserve to stressors, is associated with poor prognosis among older COVID-19 patients is unknown. The aim of our study is to investigate the association between frailty and severe disease among COVID-19 patients aged ≥ 60 years.

**Methods:**

A prospective cohort study of 114 hospitalized older patients (≥ 60 years) with confirmed COVID-19 pneumonia was conducted between 7 February 2020 and 6 April 2020. Epidemiological, demographic, clinical, laboratory, and outcome data on admission were extracted from electronic medical records. All patients were assessed for frailty on admission using the FRAIL scale, in which five components are included: fatigue, resistance, ambulation, illnesses, and loss of weight. The outcome was the development of the severe disease within 60 days. We used the Cox proportional hazards models to identify the unadjusted and adjusted associations between frailty and severe illness. The significant variables in univariable analysis were included in the adjusted model.

**Results:**

Of 114 patients, (median age, 67 years; interquartile range = 64–75 years; 57 [50%] men), 39 (34.2%), 39 (34.2%), and 36 (31.6%) were non-frail, pre-frail, and frail, respectively. During the 60 days of follow-up, 43 severe diseases occurred including eight deaths. Four of 39 (10.3%) non-frail patients, 15 of 39 (38.5%) pre-frail patients, and 24 of 36 (66.7%) frail patients progressed to severe disease. After adjustment of age, sex, body mass index, haemoglobin, white blood count, lymphocyte count, albumin, CD8+ count, D-dimer, and C-reactive protein, frailty (HR = 7.47, 95% CI 1.73–32.34, *P* = 0.007) and pre-frailty (HR = 5.01, 95% CI 1.16–21.61, *P* = 0.03) were associated with a higher hazard of severe disease than the non-frail.

**Conclusions:**

Frailty, assessed by the FRAIL scale, was associated with a higher risk of developing severe disease among older COVID-19 patients. Our findings suggested that the use of a clinician friendly assessment of frailty could help in early warning of older patients at high-risk with severe COVID-19 pneumonia.

## Background

In December 2019, cases of pneumonia with unknown origin pathogen, now known as severe acute respiratory syndrome coronavirus 2 (SARS-CoV-2), emerged in Wuhan, the capital city of Hubei Province in China [1]. The clinical manifestations of this novel disease resembled viral pneumonia [2]. The disease has rapidly spread from Wuhan to other areas in China and the World Health Organization has declared the outbreak of COVID-19 a pandemic on 11 March 2020 [3, 4]. As of 12 May 2020, over 4.0 million of confirmed COVID-19 cases and over 270 thousand deaths have been reported in over 200 countries [5].

Data from China have indicated that old age and underlying illnesses are two strong risk factors for illness and death related to COVID-19 [6–8]. Although the majority of reported COVID-19 cases were mild and the overall case-fatality rate was only 2.3%, over 80% of deaths occurred among adults aged ≥ 60 years, and the case-fatality rate increased dramatically from 3.6% among persons aged 60–69 years to 14.8% among those aged ≥ 80 years [9]. COVID-19 has also posed a disproportionately high threat to older adults in other parts of the world, including the USA and Europe [10, 11]. The COVID-19 pandemic has placed an unprecedented burden on health systems and forced health care professionals to make difficult decisions about how to allocate the increasingly scarce resources efficiently [12, 13]. In some places, decisions are being made about who should be prioritized for medical resources, such as ventilators and ICU beds, based on chronological age [14, 15]. However, chronological age may not truly reflect the differences underlying the biological ageing process and, therefore, is not an ideal basis for efficient resource allocation and establishing care plans for older COVID-19 patients. Recognizing frailty could help in early warning of older patients at high-risk with severe COVID-19 pneumonia.

Frailty is an age-related clinical syndrome of decreased reserve to stressors and is strongly associated with a wide range of adverse outcomes including death, disability, and hospitalization [16, 17]. Frailty affects over 10% of older adults in the world and its prevalence is higher at advanced age [18]. The concept of frailty and its assessment has been gradually integrated into clinical practice for evaluating prognosis and establishing goals of treatment [19]. In this sense, provision of frailty screening to older COVID-19 patients may help identify high-risk group for poor prognosis and formulate patient-tailored treatment goals. Thus, we conducted a prospective cohort study to examine whether frailty status at baseline, assessed by a clinically friendly frailty assessment, would increase the risk of development of severe disease among COVID-19 patients aged ≥ 60 years.

## Methods

### Study design and participants

We performed this prospective cohort study across two quarantine floors (wards 23 and 24) in East Campus of Renmin Hospital of Wuhan University, a hospital designated to treat COVID-19 in Wuhan, China. Each floor is equipped with an average of 40 beds. This study was approved by the Ethics Committee of West China Hospital. Because no paper documents are allowed to be taken out of the quarantine area, informed consent was obtained verbally (Y.M) from all participants.

Older patients with confirmed COVID-19 pneumonia admitted to wards 23 and 24 in the East Campus of Renmin Hospital of Wuhan University were screened for enrolment from 7 February 2020 through 6 April 2020. The inclusion criteria were as follows: (1) age ≥ 60 years, (2) diagnosis of COVID-19 pneumonia according to the WHO interim guidance published on 28 January 2020 [20], (3) completion of frailty assessment on admission (not a proxy), and (4) availability of relevant medical record information. Patients discharged within 24 h since admission were excluded.

### Assessments and outcomes

At baseline, all participants underwent assessment in 24 h, including socio-demographic data (age, sex, body mass index [BMI]), health behaviours (smoking and drinking), medical history, comorbidities (using the Charlson Comorbidity Index [21]), frailty, and laboratory data (white blood cell count, lymphocyte count, haemoglobin, albumin, creatinine, CD8+, D-dimer, C-reactive protein [CRP]). Subsequently, patients were evaluated daily until hospitalization day 60 or death/discharge via a structured interview for the development of the severe disease. We also contacted patients’ relatives by telephone to provide information about the medical history, medications, body weight, and accurate onset time of illness when the information could not be directly obtained due to low cognitive function.

Frailty was assessed on admission using the 5-item FRAIL scale, in which five criteria are included: fatigue, resistance, ambulation, illnesses, and loss of weight [22]. Patients met the criterion for fatigue if they responded “All of the time” or “Most of the time” to the question, “How much time during the past four weeks have you felt tired?”. Resistance was measured by asking patients if they had any difficulties in walking up ten steps alone without resting and without aids. Ambulation was measured by asking patients if they had any difficulty walking 100 m alone without aids. Patients met the criterion for illnesses if they reported five or more illnesses out of 11 total illnesses (hypertension, diabetes, cancer [other than a minor skin cancer], chronic lung disease, heart attack, heart failure, angina, asthma, arthritis, stroke, and kidney disease). The loss of weight criterion was met if patients reported a loss of body weight of 5% or more within the past 12 months. Frailty level was identified by the number of criteria met. Individuals with none were considered “non-frail”; those meeting one or two criteria were considered “pre-frail”, and those with three to five criteria were defined as “frail”. All participants completed the frailty assessment through self-report by themselves.

The outcome was the development of severe disease within 60 days. According to the WHO Interim guidance for COVID-19 [20], severe diseases of COVID-19 include severe pneumonia, which is defined as fever or suspected respiratory infection, plus one of the following conditions: respiratory rate > 30 breaths/min, severe respiratory distress, or SpO_2_ ≤ 93% on room air, as well as acute respiratory distress syndrome (ARDS) which is diagnosed according to the Berlin criteria [23]. Participants were censored when they were discharged or the end of the analytic period (60 days), whichever came first discharged.

To minimize error and maximize reliability, the administrator performed the following: (1) providing intensive training to the assessors including frailty and the criteria of severe disease to ensure high inter-rater reliability (kappa ≥ 0.9); and (2) all data were entered and validated by two authors to ensure reliability and accuracy.

### Statistical analysis

We presented the demographic, health behaviours, clinical, and laboratory characteristics by frailty status (non-frail, pre-frail, and frail) using medians and interquartile ranges (IQR) for continuous variables and counts and percentages for categorical variables. We compared these characteristics according to frailty status using the Kruskal-Wallis test (a nonparametric equivalent of analysis of variance) for continuous variables and chi-squared test or Fisher’s exact test for categorical variables.

**Fig. 1:**
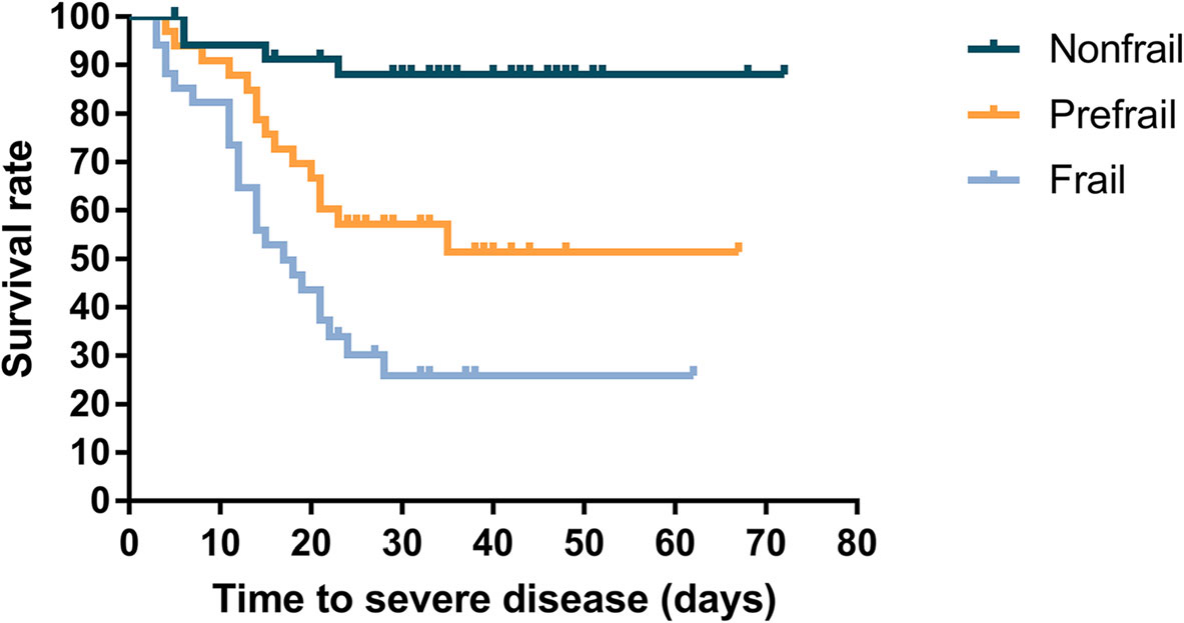
Kaplan-Meier estimates of the survival rate of severe disease among older COVID-19 patients by frailty status (non-frail, pre-frail, and frail) (non-frail and pre-frail, hazard ratio, 3.549; 95%CI, 1.016–12.396; *P* = 0.047. non-frail and frail, hazard ratio, 4.963; 95%CI, 1.443–17.074; *P* = 0.011)

We calculated the incidence rate of severe disease by frailty status. We also used the Kaplan-Meier method to estimate the survival curves of severe disease and used the log-rank (Mantel-Cox) test to compare by frailty status (Fig. 1). Subsequently, we used the Cox proportional hazards models to identify the unadjusted and adjusted associations between frailty and severe illness. The significant variables in univariable analysis were included in the adjusted model. A two-tailed α level of 0.05 was considered statistical significance for all tests. All statistical analyses were performed using SAS 9.4 (SAS Institute Inc., Cary, NC, USA).

## Results

### Sample characteristics

A total of 114 patients were included. The minimum follow-up time was 3 days and the maximum follow-up time was 55 days. The age of the patients ranged from 60 to 96 years and 50% were men. Thirty-nine (34.2%), 39 (34.2%), and 36 (31.6%) patients were classified as non-frail, pre-frail, and frail, respectively. There was no significant difference in demographic or clinical characteristics by frailty status. For laboratory characteristics, we found a steep gradient in the median level of white blood cell count, D-dimer, and CRP from non-frail to frail. Frail patients had significantly lower median level of lymphocyte count (*P* = 0.003) and CD8+ count (*P* = 0.018) than the non-frail and pre-frail (Table 1).

**Table 1:** Baseline characteristics of participants by frailty status

	Non-frail (***n*** = 39)	Pre-frail (***n*** = 39)	Frail (***n*** = 36)	***P*** value
**Characteristics**				
**Age, median (IQR), years**	67 (64–74)	68 (63–75)	73 (66–77)	0.054
**Sex, no. (%)**				0.435
Female	19 (48.72)	17 (43.59)	21 (58.33)	
Male	20 (51.28)	22 (56.41)	15 (41.67)	
**BMI, kg/m**^**2**^	23.03 (21.2–25.68)	23.04 (21.46–25.65)	23.03 (22.03–24.99)	0.720
**Smoking, no. (%)**	5 (12.82)	8 (20.51)	6 (16.67)	0.660
**Drinking, no. (%)**	6 (15.36)	5 (12.82)	6 (16.67)	0.892
**CCI**	1 (0–2)	1 (0–2)	1 (0.5–2)	0.420
**Symptoms, no. (%)**				
Fever	32 (82.05)	29 (74.36)	26 (72.22)	0.569
Myalgia	2 (5.13)	6 (15.38)	2 (5.56)	0.290
Pharyngalgia	2 (5.26)	1 (2.56)	1 (2.78)	0.843
Dry cough	28 (71.79)	22 (56.41)	22 (61.11)	0.354
Expectoration	13 (33.33)	14 (35.9)	10 (27.78)	0.747
Haemoptysis	4 (10.26)	1 (2.56)	0 (0)	0.126
Dyspnoea	11 (28.21)	16 (41.03)	19 (52.78)	0.095
Chest pain	1 (2.56)	2 (5.13)	1 (2.78)	1.000
Anorexia	20 (51.28)	24 (61.54)	25 (69.44)	0.271
Diarrhoea	6 (15.38)	2 (5.13)	4 (11.11)	0.327
Nausea	5 (12.82)	3 (7.69)	3 (8.33)	0.787
**Length of stay, median (IQR)**	17 (12–30)	25 (13–34)	27 (12–37)	0.556
**Onset-severe time, median (IQR)**	36 (29–47)	25 (16–38)	16 (11–24)	< 0.001
**Laboratory findings, median (IQR)**				
White blood cell count, × 10^9^/L	5.44 (4.21–6.77)	6.04 (4.63–8.03)	6.77 (4.53–9.7)	0.024
Lymphocyte count, × 10^9^/L	1.21 (0.79–1.7)	1.07 (0.6–1.46)	0.72 (0.53–1.21)	0.003
Haemoglobin, g/L	122 (113–132)	118 (105–130)	122.5 (111–137)	0.581
Albumin, g/L	37.4 (33.2–40.8)	36 (32.4–39.6)	36.3 (33–37.8)	0.099
**Characteristics**				
Creatinine, μmol/L	65 (53–76)	61 (53–75)	61.5 (47–75)	0.265
CD8+, count/μL	236 (160–365)	207 (120–305)	125 (44–329)	0.018
D-dimer, mg/L	0.8 (0.41–1.12)	1.61 (0.65–3.93)	1.69 (0.67–4.65)	0.004
CRP, mg/L	5.60 (0–63.5)	27.40 (0.-60.20)	55.20 (17.80–102.7)	0.008

Data are median (IQR), n (%). *p* values were calculated by Mann-Whitney *U* test, *χ*^2^ test, or Fisher’s exact test, as appropriate

*BMI* body mass index, *CRP* C-reactive protein

### Association between frailty and severe disease

During the 60 days of follow-up, 43 severe diseases occurred including eight deaths. The results of the univariate analyses were shown in the Additional file. The overall incidence rate of severe disease was 1.48 per 100 person-days (Table 2). The incidence rate of severe disease was 0.32, 1.71, and 3.73 per 100 person-days for non-frail, pre-frail, and frail patients, respectively. As shown in Table 2, 4 of 39 (10.3%) non-frail patients, 15 of 39 (38.5%) pre-frail patients, and 24 of 36 (66.7%) frail patients progressed to severe disease. The unadjusted hazard ratio of severe disease was 9.98 (95% confidence internal [CI] 3.44–29.00) among frail patients than the non-frail and 4.71 (95% CI 1.56–14.22) among the pre-frail than the non-frail (Additional file 1: Table A1). The association between frailty and severe disease persisted after adjusting for age, sex, BMI, haemoglobin, white blood count, lymphocyte count, albumin, D-Dimer, CRP, and CD8+ count. The HR of severe disease was 7.47 (95% CI 1.73–32.34) for frail patients than the non-frail. Pre-frail patients also had a significantly higher hazard of severe disease than the non-frail; the HR was 5.01 (95% CI 1.16–21.61) (Table 3). Kaplan-Meier estimates of the survival rate of severe disease among older COVID-19 patients by frailty status was showen in Fig. 1.

**Table 2:** Overall incidence rate of severe disease and incidence rate by frailty status

	Number of severe disease	Total person-days	Events per 100 person-days (95% CI)
Total (*n* = 114)	34 (29.82)	2908	1.48 (1.10–1.99)
Non-frail (*n* = 39)	4 (10.28)	1265	0.32 (0.12–0.84)
Pre-frail (*n* = 39)	15 (38.46)	879	1.71 (1.03–2.83)
Frail (*n* = 36)	24 (66.67)	643	3.73 (2.50–5.57)

*CI* confidence interval

**Table 3:** Association between frailty and severe disease

Frailty status	Minimal adjusted model*	Adjusted model^**#**^
	Hazard ratio (95% confidence interval)	
Non-frail	Ref.	Ref.
Pre-frail	4.86 (1.61–14.71)	5.01 (1.16–12.61)
Frail	10.54 (3.57–31.11)	7.47 (1.73–32.34)

*A minimally adjusted model with age and sex only

^#^Adjusted by age, sex, body mass index, haemoglobin, white blood count, lymphocyte count, albumin, CD8+ count, D-dimer, and CRP

## Discussion

We conducted a prospective cohort study of 114 older COVID-19 patients to examine the association between frailty and severe disease. We found that frailty, assessed by a self-reported frailty screening tool, was an independent risk factor for severe disease among older COVID-19 patients. These findings suggest that the FRAIL scale can be easily applied in a busy clinic setting to identify potentially severe pneumonia patients early and allows us to optimize treatment strategy in advance for older COVID-19 patients.

Since the epidemic of COVID-19, many studies have showed that older age was associated with ARDS after being infected [6, 7, 24]. According to a recent meta-analysis of community-dwelling older adults in Europe, the prevalence of physical frailty is around 15% for adults aged 65 years and over [25], and it increases to over one quarter among those over 85 years [18]. It would appear that some elderly patients still have a good prognosis, so it seems to be inappropriate to assess the prognosis and make a medical decision simply based on age. Some recent studies suggest that frailty provides better risk stratification for post-operative complications than chronological age [26, 27]. Therefore, it has been suggested that screening of frailty may be suitable for detecting older persons at increased risk of adverse outcomes [28–30]. De Silva et al. reported a positive association between frailty and mortality among nursing home residents [31]. The largest study of frailty in critical illness showed that frail adults were twice as likely to die in the hospital and within 1 year than the non-frail [32, 33]. Frailty is not only independently associated with hospitalizations and mortality in adults with pneumonia but also appears to have a synergistic effect on respiratory function along with lung disease [34, 35] Previous studies have found that age, cardiovascular disease, and cerebrovascular disease were predictive of fatal outcomes [24]. In our study, comorbidities were not associated with higher risk of severe disease in either univariate or multivariate analyses including frailty. These results suggest that frailty may better predict poor prognosis of the older patients with COVID-19 than comorbidities. Thus, we should conduct early frailty assessment, strict medical supervision, and optimal treatment for frail older patients with COVID-19, so as to improve their prognosis. Besides, it is essential to note that there is much potential for frailty to be reversed, particularly in its early stages, such as pre-frailty [36–38]. Therefore, even among the pre-frail or early frail patients with COVID-19, it is possible to improve their prognosis with early and timely management. For this reason, frailty screening should be used to detect old persons with COVID-19 for risk stratification and management guidance.

The mechanisms underlying frailty are multiple and reflect the complexity of the ageing process [35]. Theou et al. suggested that every additional year of age was associated with a 3.5 and 2.8% higher mean frailty index in lower- and higher-income countries, respectively [39]. A low-grade, persistent chronic inflammation, which is so-called inflamm-ageing, is one of the primary reason and pathobiological changes for both ageing and age-related diseases, such as frailty [35, 40]. Pro-inflammatory cytokines, including IL-6, IL-1, tumour necrosis factor (TNF)-a, C-reactive protein, and fibrinogen levels, may directly accelerate frailty by promoting muscle protein degradation [41], and the pro-inflammatory state may lead to overall suppression of the inflammatory response that is needed to fight an acute respiratory infection [42], including acute virus pneumonia such as COVID-19. However, frailty is a process with persistent chronic inflammation, whereas severe cases of COVID-19 are characterized by an inflammatory storm. Therefore, the immunologic and inflammatory mechanisms involved in the course of severe COVID-19 cases, which might be different from those involved in frailty, deserve further study. Besides, inflamm-ageing suggests a failure of the cell clearance mechanisms which could aid in the resolution of inflammation after tissue injury or/and pathogen infiltration by indirectly affecting important metabolic signalling pathways [43], which could result in the development and progression of ARDS in COVID-19 [44]. We also found that the CD8+ counts in frail COVID-19 patients were significantly lower than that in non-frail COVID-19 patients. This is consistent with the report by Liu et al., showing that the counts of lymphocyte subset (CD4+ and CD8+ T cell) are proportionally associated with disease severity [45]. Cell-mediated immune responses play an important role in virus clearance. CD4+ and CD8+ T cells are required for virus clearance during primary infection in the mucosal tissues [46]. CD8+ T cells are cytotoxic and can kill virally infected cells. Immunosenescence has been suggested to contribute to frailty and characterized by the progressive decline in both the innate and adaptive immune systems, and vice versa [42]. Viral infections usually lead to abnormal changes in the levels of lymphocyte subsets which further impaired immune system functionality [47, 48]. Therefore, frailty-related decline in immune function may explain the association between ageing and increased risk of adverse outcomes.

Our analyses were restricted to older patients with COVID-19 pneumonia, which may introduce collider bias that would undermine the validity of the observed association between frailty and progression to severe pneumonia. Selecting a sample of older patients with COVID-19 pneumonia could induce collider bias in the estimated effect of frailty on progression to severe pneumonia in the absence of adequate control for the unmeasured confounders [49]. We adjusted for several confounders including health characteristics that were associated with progression to severe pneumonia to minimize this bias. Also, simulation studies consistently showed the collider bias to only to have minimal impact on the exposure-outcome association unless the associations between some unmeasured confounders and infection of COVID-19 pneumonia were extremely large [50, 51]. Moreover, collider bias is generally thought to lead to unexpected associations, such as the paradoxical relationship between higher body mass index and better outcomes among heart failure patients [52]. We found that frail patients were more likely to experience severe disease than the non-frail; these results were consistent with the associations between frailty and poor prognostic outcomes among other populations. Therefore, the influence of collider bias on the estimate of the exposure-outcome association in the present study is not likely to be large.

Strengths of our study include that we focus on the population with COVID-19 over 60 years of age—a population that carries a disproportionally high disease burden than younger populations. Our results showed that frailty might be a risk factor for the development of severe disease in older COVID-19 patients. The FRAIL scale can be easily performed in a busy clinic setting and it will not increase the risk of the exposure of the healthcare provider to severe infectious diseases.

Our study has some limitations. First, this study was conducted at a single-centre hospital with limited sample size. A larger, multicentre cohort study of older patients with COVID-19 pneumonia would help further identify the association between frailty and prognosis. Second, all clinical and laboratory characteristics were measured once on admission. It is, therefore, challenging to distinguish confounders from mediators, which may lead to over-adjustment. Future research with repeated measures could advance our understanding about mechanisms underlying the association between frailty and poor prognosis among older COVID-19 patients. Third, the association between frailty and death was not examined in the present study. There were only eight deaths in our sample, making it difficult to study death as an outcome in a rigorous manner. Because deaths met the diagnostic criteria for then severe illness of COVID-19 and we considered death cases as “severe illness” in analyses. In the future, studies with larger sample sizes may help elucidate the relationship between frailty and mortality among COVID-19 patients. Lastly, we did not collect data on treatment options because treatment varied widely from patient to patient due to comorbidities except for antiviral regimen.

## Conclusions

Our study found that the frailty would increase the risk of development of severe disease among older COVID-19 patients—a population that carries a disproportionally high disease burden (e.g. ICU admission rate and case-fatality rate). Our findings suggested that frailty might be a predictor of poor prognosis in older COVID-19 patients. Large multicentre studies are needed to confirm our findings.

## Supplementary information

**Additional file 1: Table A1.** Univariate analysis for the severe disease.

## Data Availability

The datasets used and/or analysed during the current study are available from the corresponding author on reasonable request. Dr. Jirong Yue and Dr. Yan Kang had full access to all the data in the study and take responsibility for the integrity of the data and the accuracy of the data analysis.

## Abbreviations

ARDS: Acute respiratory distress syndrome
CCI: Charlson Comorbidity Index
COVID-19: Coronavirus disease 2019
CRP: C-reactive protein
ICU: Intensive care unit
TNF-α: Tumour necrosis factor-α
WHO: World Health Organization
