# Supplementary material for: The association between frailty and severe disease among COVID-19 patients aged over 60 years in China: a prospective cohort study
Source: BMC Med. 2020 Sep 7;18:274. doi: 10.1186/s12916-020-01761-0 (PMC7474968; doi:10.1186/s12916-020-01761-0)
Supplement: Supplementary file 1 — Additional file 1: Table A1. Univariate analysis for the severe disease. [file 12916_2020_1761_MOESM1_ESM.docx]

**Table A1. Univariate analysis for the severe disease**

| Parameter | Univariable | | 95.0% CI | |
| --- | --- | --- | --- | --- |
|  | P | HR | Lower | Upper |
| **Age** | 0.082 | 1.04 | 1.00 | 1.08 |
| **Sex** | 0.452 | 0.79 | 0.43 | 1.45 |
| **Smoking** | 0.774 | 0.88 | 0.37 | 2.09 |
| **Drinking** | 0.631 | 1.22 | 0.54 | 2.75 |
| **Hypertension** | 0.414 | 1.29 | 0.70 | 2.35 |
| **Diabetes** | 0.779 | 0.90 | 0.42 | 1.93 |
| **Coronary** | 0.091 | 2.11 | 0.89 | 5.00 |
| **Stroke** | 0.058 | 2.71 | 0.97 | 7.63 |
| **Cancer** | 0.898 | 0.93 | 0.29 | 3.00 |
| **COPD** | 0.831 | 1.11 | 0.44 | 2.82 |
| **Chronic kidney disease** | 0.098 | 2.39 | 0.85 | 6.70 |
| **Fever** | 0.242 | 0.65 | 0.32 | 1.33 |
| **Myalgia** | 0.581 | 0.72 | 0.22 | 2.32 |
| **Pharyngalgia** | 0.348 | 0.39 | 0.05 | 2.81 |
| **Cough** | 0.350 | 1.36 | 0.72 | 2.57 |
| **Expectoration** | 0.003 | 2.47 | 1.35 | 4.51 |
| **Hemoptysis** | 0.340 | 0.05 | .000 | 25.58 |
| **Dyspnea** | 0.002 | 2.63 | 1.42 | 4.87 |
| **Chest pain** | 0.762 | 0.74 | 0.10 | 5.35 |
| **Anorexia** | 0.000 | 4.38 | 1.94 | 9.86 |
| **Diarrhea** | 0.432 | 0.62 | 0.19 | 2.02 |
| **Nausea** | 0.517 | 0.68 | 0.21 | 2.19 |
| **Prefrail vs non-frail** | 0.006 | 4.71 | 1.56 | 14.22 |
| **Frail vs non-frail** | 0.000 | 9.98 | 3.44 | 29.00 |
| **BMI** | 0.110 | 0.92 | 0.84 | 1.02 |
| **CCI** | 0.144 | 1.17 | 0.95 | 1.46 |
| **WBC** | 0.000 | 1.21 | 1.12 | 1.30 |
| **Lymphocyte count** | 0.000 | 0.18 | 0.09 | 0.36 |
| **Hemoglobin** | 0.915 | 1.00 | 0.99 | 1.02 |
| **Albumin** | 0.415 | 0.97 | 0.90 | 1.04 |
| **Creatinine** | 0.707 | 1.00 | 0.99 | 1.01 |
| **CD8** | 0.005 | 1.00 | 0.99 | 1.00 |
| **D-Dimer** | 0.000 | 1.02 | 1.01 | 1.03 |
| **CRP** | 0.000 | 1.01 | 1.01 | 1.02 |
